# Nailfold capillary morphology and platelet function in patients with exfoliative glaucoma

**DOI:** 10.1371/journal.pone.0219505

**Published:** 2019-07-09

**Authors:** Vesna Maric, Anita Grgurevic, Andja Cirkovic, Sanja Stankovic, Ivan Marjanovic, Jovica Milovanovic, Andjela Milovanovic, Marija Bozic

**Affiliations:** 1 Clinic for Eye Diseases, Clinical Center of Serbia, Belgrade, Serbia; 2 Faculty of Medicine, University of Belgrade, Belgrade, Serbia; 3 Institute of Epidemiology, Faculty of Medicine, University of Belgrade, Belgrade, Serbia; 4 Department for Medical Statistics and Informatics, Faculty of Medicine, University of Belgrade, Belgrade, Serbia; 5 Center for Medical Biochemistry, Clinical Center of Serbia, Belgrade, Serbia; 6 Clinic of Otorhinolaryngology and Maxilofacial Surgery, Clinical Center of Serbia, Belgrade, Serbia; 7 Clinic for Medical Rehabilitation, Clinical Center of Serbia, Belgrade, Serbia; Oregon Health and Science University, UNITED STATES

## Abstract

**Purpose:**

The purpose of the present study was to evaluate the nailfold capillary morphological features in patients with exfoliative glaucoma (XFG) and compare them with those pertaining to primary open-angle glaucoma (POAG), normal controls and subjects with exfoliation syndrome (XFS). The second purpose was to investigate all parameters related to platelet function on the hemogram, including the platelet count (PLT), the mean platelet volume (MPV), platelet distribution width (PDW), and plateletcrit (PCT) in patients with XFG. These parameters were subsequently compared with those belonging to normal controls, POAG and XFS subjects.

**Methods:**

This case control study involved 152 consecutive patients that were examined at the Glaucoma Department of Clinic for Eye Diseases, Clinical Centre of Serbia, as the referral center for glaucoma in Serbia, between June 2016 and December 2017.

**Results:**

Regarding capillaroscopic characteristics, statistically significant difference was found in capillary diameter and tortuosity between the XFG and POAG group (p = 0.050 and p = 0.035) and the XFG and NC group (p = 0.003 and p = 0.044), as well as in the distribution of capillary loops and avascular zones between the XFG and NC group (p = 0.014 and p = 0.004). The subjects with XFG had lower PLT values compared to POAG patients (p = 0.022).

**Conclusions:**

In conclusion, to the best of our knowledge, this study marks the first attempt to evaluate capillary morphology as well as to investigate all parameters related to platelet function on the hemogram, in patients with newly diagnosed XFG. Our findings revealed nailfold capillary morphological vascular changes in XFG patients. The subjects with XFG had lower PLT values and a higher MPV serum parameter compared to normal controls and patients with POAG. Further research in this field should therefore aim to evaluate the consequences of the aforementioned microvascular abnormalities in patients with XFG.

## Introduction

Exfoliative glaucoma (XFG) is typically classified as a high-pressure type of secondary open-angle glaucoma that develops as a consequence of exfoliation syndrome (XFS) [1]. Compared with primary open-angle glaucoma (POAG), more serious clinical progression and poorer prognosis is usually associated with XFG [2,3]. XFS is a genetically and environmentally codetermined systemic condition characterized by excessive ocular and extraocular synthesis, along with progressive accumulation of a fibrillar extracellular material known as exfoliation material (XFM) [4,5,6,7]. Intraocular alterations are among the exfoliation-related clinical signs. Exfoliation material has been detected systemically, including in the vessel wall, myocardium, smooth and striated muscle cells, skin, and visceral organs [3,4,8]. Although their underlying mechanisms are not fully understood, both XFS and XFG are associated with systemic vascular diseases and abnormalities [9], such as anterior segment manifestations (iris capillary dropout), retinal diseases (retinal vein occlusion and neovascularization), systemic cardiovascular regulatory dysfunctions, and severe systemic vascular diseases [7,8,10]. Iris vasculopathy in XFS/XFG patients manifests as degeneration of the smooth muscle cells, pericytes, and endothelial cells; abnormalities of the endothelial basement membrane; and obliteration of the lumen [11,12]. Vessel lumens are often narrowed and may become obliterated, with marked alterations in the iris vasculature in advanced cases. Vessel dropout with collateral formation and iris hypoperfusion result in patchy iris microneovascularization. Fluorescein angiographic studies have shown partial occlusion of radial iris capillaries associated with hypoperfusion, a reduced number of vessels, microneovascularization, and diffuse, patchy fluorescein leakage, especially in the pupillary region [13,14]. Central retinal vein occlusions occur more frequently in XFS cases relative to controls [15,16]. Systemically, vascular changes include a lower ankle-brachial index in XFS vs. controls, which suggests that peripheral vascular disease often accompanies XFS [17]. Fingertip cutaneous capillary blood flow measured by laser Doppler flowmetry is lower, whereas the duration of cold-induced blood flow reduction as well as recovery periods are longer in XFG compared with POAG and controls [18]. However, reports of systemic vascular comorbidities in XFS/XFG remain inconsistent [10,19] and further investigations are necessary to elucidate all vascular changes in XFS/XFG.

Nailfold capillaroscopy can provide valuable information regarding the state of the vascular system at the capillary level, as well as the microvasculature at the junction of the fingernail and skin. Nailfold capillaroscopy is presently used for diagnostic purposes in rheumatology [20], while assisting in defining vascular changes in POAG and normal-tension glaucoma (NTG) [21,22,23].

In capillaroscopy, capillaries are observed through a specifically adjusted microscope, aiming to reveal the morphological and rheological (flow properties) characteristics of the capillaries. Nailfold blood vessels are easily accessible for examination due to skin transparency and their subcutaneous location. Their anatomic layout is also significant, given that their main axis at this position is parallel to the skin surface, which makes it easy to observe all capillary abnormalities. Such investigations would not be possible if some other part of the skin was subjected to capillaroscopy [24].

Although nailfold capillary hemodynamics in XFS has been previously assessed [18], there are only few published reports pertaining to the nailfold capillary morphology in XFS/XFG [25]. The nailfold capillary bed shares the same hairpin loop architecture as the iris microvasculature [26]. Hence, it may serve as a useful surrogate for gaining further insight into the systemic manifestations of XFS/XFG.

Empirical evidence indicates that an increased mean platelet volume (MPV) is associated with several vascular diseases [27]. Also, the increased MPV is higher in subjects with XFM relative to the healthy controls [28]. Additionally, the platelet count (PLT) and MPV have high specificity and predictive value for platelet activity. It has been shown that large platelets are biologically more active and have stronger prothrombotic effects [27].

The purpose of the present study was to evaluate the nailfold capillary morphological features in patients with XFG and compare them with those pertaining to POAG, normal controls and subjects with XFS. The second purpose was to investigate all parameters related to platelet function on the hemogram, including the platelet count (PLT), the mean platelet volume (MPV), platelet distribution width (PDW), and plateletcrit (PCT, total platelet mass) in patients with XFG. These parameters were subsequently compared with those belonging to normal controls, POAG and XFS subjects.

## Materials and methods

### Study population

This case control study involved 152 consecutive patients that were examined at the Glaucoma Department of Clinic for Eye Diseases, Clinical Centre of Serbia, as the referral center for glaucoma in Serbia, between June 2016 and December 2017. The sample comprised of 45 patients with newly diagnosed XFG and 40 age- and sex-matched normal controls (NC), as well as 46 patients with newly diagnosed POAG and 21 individuals with XFS. POAG was diagnosed if the clinical findings revealed the typical glaucomatous optic disc (neural rim thinning or notching, saucerization, thin nasal rim or total cupping) and/or glaucoma visual field changes, in the presence of an intraocular pressure (IOP) ≥ 22 mm Hg without medication, and a wide and open anterior chamber angle, as determined by gonioscopy assessment. Subjects were classified as having XFG if they had typical glaucomatous optic disc and/or glaucoma visual field changes in the presence of an IOP ≥ 22 mm Hg without medication, accompanied by XFM on the pupil edge and/or the anterior lens capsule after mydriasis by biomicroscopic evaluation in either or both eyes. Finally, XFS diagnosis was established by visually ascertaining presence of XFM on the pupillary margin and/or on the anterior lens surface after pupillary dilation, along with an IOP < 22 mm Hg, without evidence of glaucomatous optic nerve damage or visual field changes. XFS was established if XFM was present in either or both eyes. Patients were assigned to the normal control group if clinical examination revealed no evidence of XFM or glaucoma.

All participants were seen at our clinic on the recommendation of their primary ophthalmologist for the purpose of additional diagnostic tests and reaching a definitive glaucoma diagnosis.

Patients were excluded from the study if any of the following criteria were met: (1) use of anti-glaucoma medications; (2) use of or topical/systemic steroids; (3) previous intraocular surgery; (4) history of ocular trauma, uveitis, corneal scars, lens-induced glaucoma and any other ocular pathology that could have led to secondary glaucoma; (5) presence of autoimmune connective tissue diseases, such as systemic lupus erythematosus, systemic sclerosis, dermatomyositis, rheumatoid arthritis, and Sjogren’s syndrome, as these conditions are associated with nailfold capillary morphological abnormalities [29,30,31,32]; (6) hematological diseases and (7) manifest diabetic retinopathy, which may indicate systemic microvasculopathy [33].

All subjects that met the study inclusion criteria were given a detailed explanation of the study purpose and the nature of their involvement, and those that agreed to take part in the investigation signed an informed consent form, in accordance with the principles embodied in the Declaration of Helsinki. The study was reviewed and approved by the Ethics Committee of the Faculty of Medicine, University of Belgrade.

### Questionnaire

Participants’ demographic and comorbidity characteristics were obtained via individual interviews and by reviewing their medical documentation. The ophthalmologist (VM) performed a face-to-face interview with each patient, using a structured questionnaire, in order to obtain demographic data, medical history and information pertaining to habits relevant for the current investigation. Demographic data included age, gender and family history of glaucoma (FHG). Presence of systemic diseases, such as diabetes mellitus, diseases of the cardiovascular system in general, and specifically systemic hypertension, history of myocardial stroke, history of acute cerebrovascular disease, history of coronary artery bypass or vascular surgery, history of abdominal aortic aneurysm and antiplatelet and anticoagulant medication use, was identified through a review of medical records and during individual interviews.

Habits of interest for the study included smoking, as well as alcohol and coffee consumption. Three categories were used to describe smoking status: nonsmoker (has never smoked at least once daily for a period of 12 months), current smoker (has smoked at least once daily within the last 12 months), or ex-smoker (has previously smoked at least once daily for at least one year, but has not smoked within the preceding 12 months). Regular alcohol consumption was defined as consumption of any type of alcohol at least once daily. Similarly, regular coffee consumption was defined as coffee consumption at least once daily.

### Eye examinations

Ocular examination in all patients was performed by the same ophthalmologist (VM) and included visual acuity, slit-lamp biomicroscopy, gonioscopy (using Goldmann two-mirror indirect gonioscope), IOP measurement (using Goldmann applanation tonometry) and dilated fundus examination (using Volk Superfield +90 D lens). All subjects had daily IOP curves as a part of their medical documentation, which were taken into account when diagnosing glaucoma, in addition to the average IOP based on the three readings taken as a part of the current investigation. The mean IOP based on three readings in each eye was adopted as the pressure for that eye. A visual field test was performed using the Threshold C 24–2 Swedish Interactive Testing Algorithm (SITA) standard program with Humphrey Visual Field Analyzer II (Carl Zeiss, Germany). Visual acuity (VA) was measured by Snellen chart at 6 m distance and was converted to decimal notation, whereby the best-corrected visual acuity (BCVA) was recorded.

All normal controls and patients with POAG had their pupils dilated by administering dilation drops containing tropicamide. The same procedure was performed on patients with XFG and XFS in whom the anterior chamber angle was open. Prior to pupil dilation, a detailed high-magnification slit-lamp assessment of the pupil margin was carried out. After pupil dilation, the anterior lens surface in each eye was scanned, looking specifically for signs of XFM. If the angle was potentially occludable, the lens and the fundus evaluations were performed without dilation, and the patient was referred for a laser peripheral iridotomy. In these cases, dilated lens and fundus examinations were performed upon iridotomy completion.

Glaucoma severity indices were expressed numerically as cup to disc ratio (C/D), allowing the vertical C/D (vC/D) to be reported, along with the staging of visual filed defects using Hodapp Classification, as well as visual field mean deviation (MD). It should be noted that several patients could not partake in the visual field test due to poor visual acuity. Visual fields were evaluated by at least two experienced ophthalmologists and several perimetry measurements were obtained if required.

### Platelet parameters

After eye examinations, blood samples from each subject were collected into a serum separator tube. Blood was coagulated at room temperature for two hours and was subsequently centrifuged at approximately 1000× g for 15 minutes. Serum was separated into aliquots and was stored at -70°C until required for analyses. EDTA-anti-coagulated whole blood samples were used to obtain cell blood count by employing a new-generation automated hematology analyzer, XN-1000 (Sysmex), as it provides fluorescent platelet channel (PLT-F) that permits measurement of the fluorescent platelet count and immature platelet fraction (IPF) using only one fluorescent dye based on oxazine, which stains platelets more specifically. All analyses were carried out by the same technician who was blind to the patient’s diagnosis.

### The capillaroscopic examination

The capillaroscopic examination was performed at the Institute of Occupational Medicine of the Clinical Center of Serbia the day after eye examinations and following the administration of antiglaucoma medications. It was conducted using standard binocular microscope (Carl Zeiss, Jena, Germany) with illumination at a 45° angle, whereby blood vessels were observed at 40× magnification. The technician (GS) performing the capillaroscopy test was blind to the patient’s diagnosis. He is the only specialist in the institution where the study was conducted that is trained and qualified to perform this examination. The patient was seated during the examination with the hand at the heart level. A drop of paraffin oil was placed on the skin at the fingernail base in order to maximize the transparency of the keratin layer, whereby focusing the light beam through the microscope on the skin rendered the capillary loops visible. All the examinations were performed in the comfort zone, namely, at temperatures within the 22–25°C range, after an adaptation period of at least 15 min. Prior to the examination, the patients were instructed not to smoke for 30 min (as tobacco causes vasoconstriction), consume a large meal (owing to the changes in blood distribution following food consumption), and were not permitted to be under the influence of alcohol (due to its vasodilatatory effect). The examination was performed on the four fingers of each hand (thumbs were excluded, as it would be difficult to position the patient in a suitable position for the examination), omitting any fingers that have been affected by any type of local trauma. The most accurate data on the state of the capillaries was obtained by examining the fourth and fifth finger, due to greater skin transparency [34].

Nailfold capillaroscopy is recognized in pertinent literature as a reliable and accurate method, due to nearly perfect interobserver and intraobserver agreement (*k* = 1) [35].

The morphological characteristics of capillaries were described and rated as follows:

- Density of capillary row: within normal limits, slightly reduced, reduced
- Capillary diameter: normal, partly narrowed, narrowed, partly extended, extended
- Tortuosity: none, mild, moderate, severe
- Distribution of capillary loops along the edge of the nail plate: uniform, partly uneven, uneven
- Loop permeability: normal, lower, mildly raised, moderately raised, severely raised
- Hemorrhages: none, mild, severe
- Avascular zone

Hemorrhages were defined as extracapillary blood or hemosiderin deposits, whereas avascular zones were defined as capillary-free regions in the capillary bed that extend ≥200 μm beyond the cuticle.

### Statistical analysis

The gathered data was subjected to standard descriptive statistics. For testing the difference in frequencies between study groups Chi-squared or Fisher’s exact test was performed. The differences in numerical variables among the four groups were assessed via One-Way ANOVA combined with Tuckey post-hoc testing, or via the Kruskal-Wallis and Mann-Whitney U test. In order to identify factors associated with XFG, multivariate logistic regression was performed. Models were constructed for the following inter-group comparisons: XFG vs. POAG, XFG vs. NC, and XFG vs. XFS. The model variables were selected via the ‘enter’ method, and VIF collinearity was examined, whereby all variables with VIF > 5 were eliminated from the primary model. Results yielded by all statistical methods were significant at p < 0.05. Statistical analysis was performed in SPSS, IBM ver. 24.0.

## Results

The subjects’ demographic characteristics and their habits, as well as the prevalence of systemic diseases in the sample, are shown in Table 1.

**Table 1:** Demographic characteristics, systemic diseases and habits of subjects with XFG, POAG, XFS and NC.

Characteristic	XFG (N = 45)	POAG (N = 46)	p^b^	NC (N = 40)	p^c^	XFS (N = 21)	p^d^	p^a^
Age, mean±SD	73.0±9.1	66.1±8.1	0.001*	73.0±8.2	1.000	74.5±6.5	0.905	<0.001*†
Male gender, n (%)	31 (68.9)	24 (52.5)	0.103	28 (70.0)	0.912	11 (52.4)	0.194	0.196¥
DM, n (%)	12 (26.7)	14 (30.4)	0.691	11 (27.5)	0.931	6 (28.6)	0.871	0.981¥
CVD, n (%)	36 (80.0)	34 (73.9)	0.491	28 (70.0)	0.286	16 (76.2)	0.724	0.758¥
SH, n (%)	34 (75.6)	30 (65.2)	0.153	28 (70.0)	0.565	16 (76.2)	0.955	0.683¥
MS, n (%)	6 (13.3)	2 (4.3)	0.130	3 (7.5)	0.383	2 (9.5)	0.659	0.487‡
CAB or VS, n (%)	9 (20.0)	4 (8.7)	0.123	3 (7.5)	0.099	3 (14.3)	0.575	0.273‡
AAA, n (%)	3 (6.7)	1 (2.2)	0.296	0 (0)	0.096	1 (4.8)	0.763	0.345‡
ACD, n (%)	4 (8.9)	2 (4.3)	0.383	3 (7.5)	0.816	1 (4.8)	0.555	0.859‡
Antiplatelet and anticoagulant m (Acidum acetylsalicylicum, apixaban, rivaroxaban), n (%)	32 (71.1)	33 (71.7)	0.947	26 (65.0)	0.546	16 (76.2)	0.666	0.814¥
Smoking, n (%)			0.182		0.136		0.669	0.333¥
Non-smoker	21 (46.7)	23 (50.0)		25 (62.5)		12 (57.1)		
Ex-smoker	14 (31.1)	19 (41.3)		12 (30.0)		6 (28.6)		
Current	10 (22.2)	4 (8.7)		3 (7.5)		3 (14.3)		
Alcohol, n (%)	13 (28.9)	16 (34.8)	0.546	16 (40.0)	0.281	8 (38.1)	0.455	0.737¥
Coffee, n (%)	39 (86.7)	39 (84.8)	0.797	30 (75.0)	0.170	16 (76.2)	0.287	0.449¥
FHG, n (%)	6 (13.3)	17 (36.9)	0.010	2 (5.0)	0.189	2 (9.5)	0.659	0.001¥

XFG: exfoliative glaucoma; POAG: primary open-angle glaucoma; NC: normal controls; XFS: controls with exfoliation syndrome; N: number of subjects; y: years; DM: diabetes mellitus; CVD: cardiovascular diseases; SH: systemic hypertension; MS: myocardial stroke; CAB: coronary artery bypass; VS: vascular surgery; AAA: abdominal aortic aneurysm; ACD: acute cerebrovascular disease; FHG—family history of glaucoma

*Statistically significant p values.

^a^between all groups

^b^XFG vs. POAG

^c^XFG vs. NC

^d^XFG vs. XFS

†One Way ANOVA with Tuckey posthoc

¥Chi-square test

‡Fisher`s exact test

With regard to systemic diseases, there were no statistically significant differences among groups. As shown in Table 1, compared with other groups, a greater percentage of patients in the XFG group reported history of abdominal aortic aneurysm, as well as coronary artery bypass or vascular surgery; however, the difference was borderline significant for XFG vs. NC only (6.7% vs. 0%, p = 0.096 and 20.0% vs. 7.5%, p = 0.099). Moreover, compared with the POAG patients, a greater percentage of XFG patients reported a negative FHG (86.7% vs. 63.1%, p = 0.010).

The examination was performed on 304 eyes, 71 (23.3%) of which pertained to XFG, 92 (30.3%) to POAG, and XFS was noted in 41 (13.5%) eyes, while in the remaining 100 (32.9%) eyes served as normal control group, owing to the absence of XFS or any type of glaucoma. Cataract percentages for XFG, POAG, NC and XFS groups were 74.6%, 26.1%, 56.0% and 75.6%, respectively, p < 0.001.

Glaucoma severity indices obtained for the XFG and POAG eyes at the first examination are shown in Table 2.

**Table 2:** Clinical features and indices of glaucoma severity in XFG and POAG eyes at presentation.

Characteristic	XFG (N = 71)	POAG (N = 92)	p
BCVA			0.001*§
med (min–max)	0.7 (0.008–1.0)	1.0 (0.02–1.0)	
LP, n (%)	3 (4.2)	0 (0)	
NLP, n (%)	2 (2.8)	0 (0)	
IOP (mmHg), mean±SD	32.1±9.8	28.1±4.8	<0.001*†
Hodapp, n (%)			0.058¥
early	29 (40.8)	53 (57.6)	
moderate	10 (14.1)	16 (17.4)	
advanced	22 (31.0)	17 (18.5)	
without visual field	10 (14.1)	6 (6.5)	
MD, med (min-max)	-5.26 (-1.58 to -28.27)	-4.35 (-1.39 to -31.02)	0.034*#
vC/D, med (min-max)	0.6 (0.45–1.0)	0.5 (0.45–1.0)	0.001*#

BCVA: best-corrected visual acuity; LP: light perception; NLP: no light perception; IOP: intraocular pressure; MD: mean deviation; vC/D: vertical cup to disc ratio; N: number of eyes

*Statistically significant p value

†One Way ANOVA with Tuckey posthoc

§ Kruskal-Wallis test with Mann Whitey U test afterwards

¥Chi-square test

# Mann Whitney test

Regarding capillaroscopic characteristics, statistically significant difference was found in capillary diameter and tortuosity between the XFG and POAG group (p = 0.050 and p = 0.035) and the XFG and NC group (p = 0.003 and p = 0.044), as well as in the distribution of capillary loops and avascular zones between the XFG and NC group (p = 0.014 and p = 0.004) (Table 3).

**Table 3:** Capillaroscopy characteristics of subjects with XFG, POAG, XFS and NC.

Characteristic, n (%)	XFG (N = 45)	POAG (N = 46)	p^b^	NC (N = 40)	p^c^	XFS (N = 21)	p^d^	p^a^
**Capillary row density**			0.271		0.074		0.100	0.129¥
Within normal limits	15 (33.3)	21 (45.7)		23 (57.5)		12 (57.2)		
Slightly reduced	23 (51.1)	22 (47.8)		14 (35.0)		5 (23.8)		
Reduced	7 (15.6)	3 (6.5)		3 (7.5)		4 (19.0)		
**Capillary diameter**			0.050*		0.003*		0.678	0.035*¥
Normal	5 (11.1)	14 (30.4)		17 (42.5)		4 (19.0)		
Partly narrowed	19 (42.2)	20 (43.5)		16 (40.0)		10 (47.7)		
Narrowed	8 (17.8)	5 (10.9)		3 (7.5)		4 (19.0)		
Partly extended	6 (13.3)	6 (13.0)		4 (10)		2 (9.5)		
Extended	7 (15.6)	1 (2.2)		0 (0)		1 (4.8)		
**Tortuosity**			0.035*		0.044*		0.917	0.126¥
None	5 (11.1)	10 (21.8)		10 (25)		3 (14.3)		
Mild	20 (44.4)	28 (60.9)		23 (57.5)		10 (47.7)		
Moderate	12 (26.7)	6 (13.0)		5 (12.5)		4 (19.0)		
Severe	8 (17.8)	2 (4.3)		2 (5.0)		4 (19.0)		
**Distribution of capillary loops**			0.130		0.014*		0.133	0.015*¥
Uniform	16 (35.6)	18 (39.1)		26 (65.0)		13 (61.9)		
Partly uneven	11 (24.4)	18 (39.1)		8 (20.0)		3 (14.3)		
Uneven	18 (40)	10 (21.8)		6 (15.0)		5 (23.8)		
**Loop permeability**			0.239		0.075		0.021*	0.106¥
Normal	14 (31.1)	21 (45.6)		21 (52.5)		13 (61.9)		
Mildly raised	18 (40)	17 (37.0)		14 (35.0)		2 (9.5)		
Moderately raised	13 (28.9)	7 (15.2)		5 (12.5)		6 (28.6)		
Lower	0 (0)	1 (2.2)		0 (0)		0 (0)		
**Hemorrhages**			0.126		0.054		0.207	0.060¥
None	25 (55.6)	27 (58.7)		27 (67.5)		9 (42.8)		
Mild	14 (31.1)	18 (39.1)		13 (32.5)		11 (52.4)		
Severe	6 (13.3)	1 (2.2)		0 (0)		1 (4.8)		
**Avascular zones**			0.947		0.004*		0.979	0.024*¥
Yes	13 (28.9)	13 (28.3)		2 (5.0)		6 (28.6)		
No	32 (71.1)	33 (71.7)		38 (95.0)		15 (71.4)		

N*: number of subjects

^a^between all groups

^b^XFG vs. POAG

^c^XFG vs. NC

^d^XFG vs. XFS

*Statistically significant p values.

¥Chi-square test

Table 4 shows a comparison of the parameters related to platelet function on the hemogram, including PLT, MPV, PDW and PCT results between patients with XFG and those with POAG, normal controls and XFS subjects.

**Table 4:** Comparison of the platelet function between subjects with XFG, POAG, XFS and NC.

Parameters	XFG	POAG	p^b^	NC	p^c^	XFS	p^d^	p^a^
PLT	218.30±46.28	249.58±58.52	0.022*	232.98±51.23	0.373	235.28±70.68	0.446	0.040*†
MPV	10.10±1.43	9.55±1.78	0.283	9.41±1.29	0.088	9.72±1.34	0.736	0.113†
PCT	0.223±0.063	0.243±0.065	0.333	0.220±0.054	0.987	0.227±0.071	0.992	0.194†
PDW	18.69±12.83	17.04±11.089	0.857	16.61±5.71	0.688	15.11±2.63	0.436	0.472†

PLT: the platelet count; MPV: the mean platelet volume, PDW: platelet distribution width; PCT: plateletcrit

^a^between all groups

^b^XFG vs. POAG

^c^XFG vs. NC

^d^XFG vs. XFS

*Statistically significant p values.

†One Way ANOVA with Tuckey posthoc

When the participants in the XFM (XFG and XFS) group were compared to subjects without XFM (those assigned to the POAG and NC groups), their serum PLT and MPV parameters where statistically significantly different, as indicated in Table 5.

**Table 5:** Comparison of the platelet function between subjects with XFM (XFG/XFS) and non-XFM (NC/POAG).

Parameters	XFM	Non XFM	p
PLT	222.14±58.43	240.11±54.89	0.041*≠
MPV	9.98±1.40	9.47±1.52	0.030*≠
PCT	0.224±0.065	0.231±0.060	0.529≠
PDW	17.48±10.65	16.81±8.54	0.796≠

PLT: the platelet count; MPV: the mean platelet volume

PDW: platelet distribution width; PCT: plateletcrit

*Statistically significant p values.

≠student`s t test

Factors associated with XFG that, according to the multivariate logistic regression analysis results, distinguish it from POAG, NC and XFS are shown in Table 6.

**Table 6:** Factors associated with XFG vs. POAG, NC, XFS according to multivariate logistic regression analysis.

	OR, 95% CI OR	p^a^	p^b^	p^c^
XFG vs. POAG				
IOP	0.994, 0.93–1.07	0.879	0.925	0.945
BCVA	7.734, 1.28–46.84	0.026*	0.081	0.072
vC/D	13.631, 1.21–153.0	0.034*	0.082	0.100
MD	0.746, 0.53–1.04	0.085	0.075	0.010*
Tortuosity	0.697, 0.48–1.01	0.059	0.077	0.033*
Capillary diameter	0.853, 0.63–1.15	0.304	0.510	0.512
PLT	1.011, 1.00–1.02	0.005*	0.007*	0.002*
XFG vs. NC				
IOP	0.628, 0.49–0.80	<0.001*	0.001*	0.001*
BCVA	1.556, 0.02–100.38	0.835	0.753	0.729
MD	0.188, 0.04–0.92	0.039*	0.033*	0.036*
Capillary diameter	1.133, 0.67–1.93	0.645	0.938	0.846
Avascular zones	10.644, 1.79–63.37	0.009*	0.031*	0.038*
Distribution of capillary loops	0.507,0.22–1.18	0.116	0.080	0.084
Tortuosity	0.619, 0.30–1.25	0.183	0.503	0.444
XFG vs. XFS				
IOP	0.575, 0.43–0.77	<0.001*	0.001*	0.001*
BCVA	0.096, 0.01–2.74	0.171	0.164	0.346
vC/D	1.095, 0.40–3.01	0.860	0.505	0.631
MD	1.357, 0.58–3.19	0.485	0.587	0.599
Permeability	0.537, 0.28–1.03	0.062	0.185	0.198

IOP: intraocular pressure; BCVA: best-corrected visual acuity; vC/D: vertical cup to disc ratio; MD: mean deviation; PLT: platelet count

*Statistically significant p values.

^a^: adjusted for age and gender

^b^: adjusted for age, gender, presence of diabetes mellitus and cardiovascular diseases, smoking, alcohol consumption, use of antiplatelet and anticoagulant medication

^c^: adjusted for age, gender, smoking, alcohol consumption, use of antiplatelet and anticoagulant medication, coronary artery bypass and vascular surgery, systemic hypertension, myocardial stroke, abdominal aortic aneurysm and acute cerebrovascular disease

## Discussion

Both XFS and XFG are associated with systemic vascular diseases and abnormalities [8,9]. Although no uniform relationship between XFS/XFG and clinical systemic vascular diseases has been established across diverse populations, vascular dysfunction, irrespective of the degree of clinically significant consequences, has been repeatedly reported in studies involving both epidemiological and pathophysiological methods [9].

Although the systemic vascular abnormalities in XFS and XFG have been extensively studied, according to an extensive literature search, there is a paucity of studies focusing on nailfold capillary morphology in patients with XFS and XFG [25], and their characteristics have never been compared as a part of a single investigation. In particular, to the best of our knowledge, this is the first attempt to investigate capillary morphology in patients with a newly diagnosed XFG.

The density of the capillary row in healthy persons increases with age, partially due to the natural maturation process [36]. Hence, reduced capillary row density is a pathological finding indicative of tissue hypoxia, and is often found in patients affected by systemic diseases (e.g., systemic sclerosis, dermatomyositis, etc.). In the sample examined as a part of the present study, no statistically significant differences in the capillary row density were noted among the XFG, POAG, NC and XFS groups. In their study, Bozic et al. [23] also conducted nailfold capillaroscopy using the same method and capillaroscopy protocol as those adopted in the present investigation. However, their sample comprised of patients with POAG and normotensive glaucoma (NTG). These authors found no statistically significant differences in the capillary row density between the POAG and NTG patients. While we also assessed the capillary diameter, it should be noted that there is no universal rating method for this parameter. According to the Maricq method, which is the most frequently adopted approach, 25−50 μm is the upper limit for the capillary diameter in adults [37]. In our study, we found a statistically significant difference in this capillaroscopic characteristic between XFG and NC as well as POAG patients. Conversely, Bozic et al. reported no statistically significant differences in the capillary diameter, but the comparison was made between POAG and NTG subjects. In the present study, in the XFG group, a greater number of patients had narrow and partly narrowed capillaries relative to the other groups. As noted earlier, the nailfold capillary bed shares the same hairpin loop architecture as the iris microvasculature [26]. Iris vessel lumens are often narrowed and may become obliterated, with marked alteration of the iris vasculature in advanced cases. The XFG group also had the greatest percentage of cases with uneven capillary loop distribution, whereby the difference was statistically significant between the XFG and NC group only. The capillary loop resistance and permeability reflect the state of the capillary wall. Increased capillary permeability is considered a result of advanced vascular changes, such as those caused by bursting of the capillary wall, which in turn modifies blood flow through the capillary. Increased, and in particular mildly elevated permeability, was more frequently noted in the XFG group, with a statistically significant difference between the XFG and XFS group and a borderline significant difference relative to the POAG group. It should be noted, this is the only capillaroscopic characteristic with a difference between the XFG and XFS groups. A greater percentage of severe hemorrhages was also noted in the XFG group relative to the other three, with a borderline significant difference compared to the NC group. In addition, the XFG group was characterized by a statistically significantly higher degree of tortuosity (particularly moderate and severe tortuosity) compared to the POAG and NC groups. We also found a stronger association between the presence of avascular zones and XFG compared with normal controls. Analysis of capillaroscopic findings revealed statistically significant differences in most of the investigated characteristics between the group of patients with XFG and those assigned to the NC group, while the differences between the XFG and XFS group were not statistically significant, indicating that vascular abnormalities are present in XFG/XFS. These findings represent a non-ocular feature of XFS/XFG and demonstrate that non-ocular microvascular changes accompanying XFS/XFG may be conveniently observed in the clinic.

Vascular dysfunction in XFS/XFG comprises anatomic alterations of the vessel wall because of elastosis and elevated serum homocysteine (e.g., venous occlusions and aortic aneurysms), and alterations that are predominantly caused by systemic vascular dysregulation (impaired baroreflex regulation [4], decreased carotid artery distensibility and increased carotid artery stiffness [4], impaired conduit artery function [4], reduced cutaneous capillary reactions [18], severely pathologic heart rate variability indices [38] and impaired vascular endothelial function [39]).

The processes by which nailfold capillary abnormalities develop in XFG remain unknown. Hence, the following discussion will focus on some plausible reasons behind this phenomenon. Accumulation of exfoliation material in the vessel wall results in degenerative vascular changes [3], while XFM accumulation in the walls of digital precapillary arterioles or in nailfold capillaries produces nontubular vascular lumens that alter local hemodynamics and contribute to the morphological abnormalities observed in this study. Furthermore, increased oxidative stress and decreased antioxidant protection, together with the XFS/XFG-related elastosis, may play a role in the development of vascular dysfunction. Insufficient elastogenesis due to altered lysyl oxidase-like 1 (LOXL1) expression [5,40,41] may contribute to microvascular morphological changes. LOXL1 is a key enzyme involved in elastic fiber synthesis and homeostasis, exhibiting its role by catalyzing the covalent cross-linking of tropoelastin monomers into elastin polymers [41]. Genetic studies in multiple populations have provided conclusive evidence that single nucleotide polymorphisms (SNPs) in exon 1 of the LOXL1 gene represent the principal genetic risk factor for both XFS and XFG [42,43].

However, it is also hypothesized that elevated plasma homocysteine level can be another contributing factor to the nailfold capillary abnormalities. Available evidence indicates that homocysteine (Hcy) is a biomarker that is elevated in XFG and XFS patients [44]. Elevated Hcy is associated with endothelial cell dysfunction, in particular in the impaired endothelial dependent dilatation aspect [45]. As proposed by Cousins et al. [25], abnormal endothelial cell function due to elevated Hcy may influence vascular tone regulation in XFS/XFG, rendering the nailfold capillaries tortuous and prone to leakage.

The more frequent presence of nailfold hemorrhages in XFG, POAG and XFS in our study (44.4%, 41.3% and 57.1%) relative to normal controls (32.5%) is in line with the findings reported by Cousins et al. [25], who speculated that impaired nitric oxide signaling might be responsible for nailfold hemorrhages. They supported this assertion by citing studies [46,47] whose authors demonstrated that asymmetric dimethyl arginine (ADMA), an inhibitor of nitric oxide synthase and contributor to endothelial cell dysfunction, is elevated in the aqueous humor and serum of XFS/XFG patients. However, Cousins et al. [25] assessed nailfold capillary morphological features in XFS/XFG as a single group, whereas these patients were segregated into two groups in our study and were compared with either POAG cases or normal controls. These authors found that nailfold capillary hemorrhages, avascular zones, and degree of microvascular tortuosity, as individually investigated features, had a greater association with XFS/XFG and POAG than with normal controls. While nailfold capillary morphology was the focus of the present study, Philip et al. [48] recently investigated the peripheral nailfold blood flow circulation in XFG, concluding as we did that the vascular morphological and hemodynamic alterations in the peripheral nailfold circulation in XFG may serve as evidence of the systemic nature of exfoliation glaucoma. In their study, Philip et al. examined not only patients with XFG, but also individuals diagnosed with normal-tension glaucoma (NTG). Both groups showed decreased peripheral blood flow at the nailfold of the fourth digit compared with normal control participants. However, patients in the XFG group had a lower and limited range of resting peripheral blood flow compared with the NTG group, high-tension group (HTG) and normal controls. The authors posited that this limited range may represent stiffened vasculature or impaired maximal flow in the peripheral vasculature of patients with XFG. The limited range may also be associated with increased tortuosity in patients with XFS/XFG, as previously described by Cousins et al. [25]. Philip et al. [48] further argued that the pathophysiological mechanisms behind these flow alterations may be linked to the accumulation of exfoliation material in tissue, causing degenerative fibrillopathy in normal basement cell membranes. This degeneration may cause the aforementioned alterations in the peripheral vessels, including elastosis, oxidative stress, and vascular endothelial dysfunction, leading to vascular stiffness [9]. Similar results related to lower peripheral blood flow in patients with HFG and NC were reported by Cousins et al. [49], who explored resting peripheral blood flow at the nailfold in patients with POAG (HTG and NTG), which was found to be significantly lower compared with that in normal controls.

It is important to emphasize that other, currently unknown, mechanisms may also play an important role in the development of vascular dysfunction in XFS/XFG, and a combination of different mechanisms is highly probable. However, it should be noted that plasma homocysteine and ADMA levels were not measured in the present study. Thus, further research is necessary to evaluate other potential mechanisms contributing to the development of vascular abnormalities in patients with XFG.

In order to provide evidence of such alternative mechanisms, we examined patients’ blood counts, with a particular emphasis on parameters related to platelet function on the hemogram. Platelets’ main function is to contribute to hemostasis as a blood component. Platelets represent an important link between inflammation, thrombosis and atherogenesis [50] and have an important role in the initiation of atherosclerotic lesions and subsequent complications [51]. Platelets in the blood of each individual are heterogeneous in size and density, whereby their size can impact their functions [52]. Mean platelet volume (MPV), as the most commonly used measure of platelet size, can be useful for predicting platelet functional changes and activation patterns [53]. MPV, PDW and other complete blood counts can be easily evaluated by routine hematological analyzers. Empirical evidence indicates that MPV is increased in various cardiovascular diseases, as well as in peripheral artery and cerebrovascular diseases [27,54]. In addition, in subjects with established cardiovascular disease, elevated MPV may be a marker for adverse cardiovascular events. The relationship between MPV and the number of platelets is, however, unclear. Authors of several studies have reported that increases in platelet volume are often associated with decreases in platelet count, perhaps as a result of small platelets being consumed in order to maintain a constant platelet functional mass [27,55]. In our study, the PLT values measured for the XFG group were statistically significantly lower than those in the control group, and especially compared to the POAG subjects. It should be noted that, while using antiplatelet or anticoagulation medication may influence platelet count, no statistically significant differences among the studied groups (and especially between the XFG and POAG patients) were noted with respect to medication use. On the other hand, when in the multivariate logistic regression analysis we adjusted for antiplatelet or anticoagulation medication use, a stronger association was noted between the PLT and XFG compared with the POAG group. When the participants in the XFM (XFG and XFS) group were compared to subjects without XFM (those assigned to the POAG and NC groups), their serum PLT and MPV parameters where statistically significantly different. Our findings counter those reported by Yazgan et al. [56] who obtained higher PLT values in the XFM group relative to the non-XFM group, but the difference was not statistically significant, while reporting borderline differences in the MPV parameter between the XFM and non-XFM group, whose PDW and PDC values were significantly different. On the other hand, our findings are supported by the results obtained by Türkcü et al. [28], who found that the MPV values in both XFM groups were higher than those in the normal control group.

Systemic vascular alterations seem to develop and then progress with the duration of clinically manifested XFS/XFG [8]. In our study, nailfold capillaroscopy revealed microvascular abnormalities in XFG patients compared to normal controls, as well as differences in PLT values between the XFG and POAG subjects. Moreover, assessment of the prevalence of clinical systemic vascular diseases failed to reveal any statistically significant differences between XFG and other studied groups with respect to systemic hypertension, history of myocardial stroke, history of acute cerebrovascular disease, history of abdominal aortic aneurysm, and history of coronary artery bypass or vascular surgery.

Nonetheless, it needs to be emphasized that systemic diseases were not clinically determined in our study sample. Instead, the existence of systemic diseases was established via interviews, as well as through an overview of medical documentation, which could be considered one of the study limitations. Given that this was a case-control study, it is essential to recognize the limitations of this research design, in particular this approach pertains to the inability to provide a direct estimate of risk (relative risk or risk ratio), due to which we have only calculated and reported the odds ratio. Moreover, while recall bias is almost always a limitation in a case-control study design, it was minimized in this work by conducting a detailed medical history review. As this was not a population-based study, no conclusion on the population-level differences can be drawn from the results. As a further study limitation, we were unable to determine whether nailfold capillary morphological changes precede or succeed the development of XFM in the eye. It is also noteworthy that we might have failed to detect some subclinical cases of XFS that would be diagnosable by histological methods only. Specifically, as determining the age at which XFG and POAG commonly occurs was the main study aim, POAG patients included in the study sample were not age- or sex-matched with those included in the XFG group.

It should also be noted that XFG clinical features are distinct from those characterizing POAG at the time of diagnosis. Compared with the POAG group, higher mean IOP was measured in the XFG group. Moreover, a significantly greater severity of optic nerve damage was noted in newly diagnosed XFG compared to newly diagnosed POAG, as indicated by the vC/D values. In addition, the newly diagnosed XFG patients also had significantly more advanced visual field changes than those newly diagnosed with POAG.

## Conclusions

In conclusion, to the best of our knowledge, this study marks the first attempt to evaluate capillary morphology as well as to investigate all parameters related to platelet function on the hemogram, in patients with newly diagnosed XFG. Our findings revealed nailfold capillary morphological vascular changes in XFG patients. However, even though nailfold capillaroscopy revealed microvascular abnormalities in XFG patients, no association between clinical systemic vascular diseases and XFG was established. The subjects with XFG had lower PLT values and a higher MPV serum parameter compared to normal controls and patients with POAG. Further research in this field should therefore aim to evaluate the consequences of the aforementioned microvascular abnormalities in patients with XFG.

## Supporting information

S1 Database This is S1 database raw data. Legend: The raw data of this study, such as demographic data, comorbidity data, ophthalmic characteristics, the morphological characteristics of capillaries and platelet parameters for each subject separately are shown in the SPSS database.
(SAV)
